# Area-Wide Ground Applications of *Bacillus thuringiensis* var. *israelensis* for the Control of *Aedes albopictus* in Residential Neighborhoods: From Optimization to Operation

**DOI:** 10.1371/journal.pone.0110035

**Published:** 2014-10-20

**Authors:** Gregory M. Williams, Ary Faraji, Isik Unlu, Sean P. Healy, Muhammad Farooq, Randy Gaugler, George Hamilton, Dina M. Fonseca

**Affiliations:** 1 Hudson Regional Health Commission, Secaucus, New Jersey, United States of America; 2 Center for Vector Biology, Rutgers University, New Brunswick, New Jersey, United States of America; 3 Salt Lake City Mosquito Abatement District, Salt Lake City, Utah, United States of America; 4 Mercer County Mosquito Control, West Trenton, New Jersey, United States of America; 5 Department of Pathobiological Sciences, Louisiana State University School of Veterinary Medicine, Baton Rouge, Louisiana, United States of America; 6 Navy Entomology Center of Excellence, Jacksonville, Florida, United States of America; University of Queensland & CSIRO Biosecurity Flagship, Australia

## Abstract

The increasing range of *Aedes albopictus*, the Asian tiger mosquito, in the USA and the threat of chikungunya and dengue outbreaks vectored by this species have necessitated novel approaches to control this peridomestic mosquito. Conventional methods such as adulticiding provide temporary relief, but fail to manage this pest on a sustained basis. We explored the use of cold aerosol foggers and misting machines for area-wide applications of *Bacillus thuringiensis* var. *israelensis* (VectoBac WDG) as a larvicide targeting *Aedes albopictus*. During 2010–2013 we performed initially open field trials and then 19 operational area-wide applications in urban and suburban residential areas in northeastern USA to test three truck-mounted sprayers at two application rates. Area-wide applications of WDG in open field conditions at 400 and 800 g/ha killed on average 87% of tested larvae. Once techniques were optimized in residential areas, applications with a Buffalo Turbine Mist Sprayer at a rate of 800 g/ha, the best combination, consistently provided over 90% mortality. Importantly, there was no significant decrease in efficacy with distance from the spray line even in blocks of row homes with trees and bushes in the backyards. Under laboratory conditions *Bti* deposition in bioassay cups during the operational trials resulted in over 6 weeks of residual control. Our results demonstrate that area-wide truck mounted applications of WDG can effectively suppress *Ae. albopictus* larvae and should be used in integrated mosquito management approaches to control this nuisance pest and disease vector.

## Introduction

The range of *Aedes albopictus*, the Asian tiger mosquito, is expected to expand in highly urbanized temperate regions in North America and Europe in response to climate change [1]–[3]. An aggressive human biter, this mosquito is often the primary pest species eliciting complaints from the public in areas where it occurs [4] and it also presents a significant health risk [1], [5], [6]. Unfortunately, conventional mosquito abatement methods do not control *Ae. albopictus* effectively. Local mosquito control agencies across the US mount aggressive control campaigns against salt marsh, floodwater, and many other rural-based mosquito pest species [2]. Since the onset of the WNV epidemic mosquito control agencies in urban and suburban areas have targeted *Culex* spp. in residential storm drains and other large stagnant water sources [2]. These programs rarely impact *Ae. albopictus*, which thrive in small pockets of water in artificial containers such as buckets, toys and trash primarily within private yards [7]. Therefore, efforts tend to be limited to public education, placing the responsibility for control on unqualified citizens [8], [9]. Having individual homeowners bear considerable responsibility for source reduction has hampered control efforts primarily because residents are not usually able to coordinate their efforts with enough of their neighbors to eliminate the larval sources that contribute biting *Ae. albopictus* to a neighborhood.

For any significant suppression, this pest must be managed on an area-wide basis [9]. Intensive source reduction efforts which involve teams of professionals emptying, removing or treating all sources of standing water in thousands of private yards can be effective. However, they are extremely labor intensive leading to unsustainably high costs [9]–[11] and often also temporary since new containers are continuously being added by uninformed homeowners [7], [12]. Area-wide efforts require relatively swift and repeatable applications of insecticides at a large scale and low volume usually from a ground vehicle or aircraft.

Although *Ae. albopictus* is a day-biting mosquito, adulticide applications at night when thermal inversions optimize likelihood of insecticide deposition, non-target species are mostly inactive and residents are less likely to be outside, can be effective [13]. Unfortunately, results are temporary [13] because such methods kill adults without addressing the large pool of immature stages that continuously emerge into adults.

Area-wide application of mosquito larvicides involves applying liquid or emulsified larvicides with a low-volume sprayer so the droplets drift onto backyards. The droplets of larvicide then settle into the myriad of containers of water where *Ae. albopictus* can be found, delivering a lethal dose. Using this approach hundreds of residential yards can be treated in a single application during one night, an action that can be repeated as needed, as opposed to intensive source reduction approaches that would take minimally weeks to treat the same number of yards [9].

*Bacillus thuringiensis* subspecies *israelensis* (*Bti*) is an excellent candidate for a low-volume area-wide strategy. The efficacy of *Bti* has been demonstrated for many mosquito species in a variety of habitats. Today, formulations of *Bti* are one of the primary products used for larval mosquito control in the U.S. and other countries [14]. After ingestion and activation in the mosquito larvae, which have a highly basic gut pH, proteinaceous toxins are released which cause lysis of the epithelial cells of the larval midgut, killing the larvae. *Bti* contains four different larvicidal proteins, each acting in slightly different ways making the development of resistance to *Bti* difficult [15]. The specificity of these proteins to organisms with catalytic gut pH results in very few non-target impacts. With the exception of several families of Nematocera (many of them also pest species such as blackflies and biting midges), *Bti* has no known effects on other insects, invertebrates, or vertebrates [16].

Although *Bti* has often been applied as a solid directly to larval sources, recent studies have demonstrated that *Bti* liquid can be applied as ULV or low volume (LV) sprays to control mosquitoes. Several such studies have successfully used the VectoBac 12AS formulation (Valent BioSciences Corporation, Libertyville, IL, USA) [17]–[19], however 12AS can cause persistent spotting on automotive paint [20], [21] and is therefore unsuitable for use in residential areas in the US.

Until now, studies that used the VectoBac WDG formulation (Valent BioSciences Corporation, Libertyville, IL, USA) focused on backpack mist sprayers that are insufficient for area-wide control efforts because they hold small volumes of product and require the operator to walk into backyards during the application [17], [22], [23]. These manual applications are problematic because it takes too much time to treat a neighborhood and access to yards is often restricted by fences, dogs, or uncooperative homeowners. Most studies were conducted in Southeast Asia where differences in the strains of *Ae. albopictus*, housing construction and condition, and accepted pest management practices limit the value of those data in the US. The present study describes the development of an area-wide larviciding strategy using VectoBac WDG applied with truck-mounted equipment. We evaluated several sprayers and application rates under both staged open-field as well as operational trials in urban and suburban settings, and used larval bioassays to examine the efficacy of this method over distance (from the road into the backyards) and over time (residual effects). Our ultimate goal is to develop a novel strategy for the control of *Ae. albopictus* larvae to be used in an integrated mosquito management program in hopes of reducing the nuisance and disease risk associated with this species.

## Materials and Methods

### Ethics Statement

No specific permits were required for the described field studies. All pesticide applications were made by county mosquito control agencies under the authority of Title 26∶9 of the New Jersey Administrative Code. These studies did not involve endangered or protected species.

### Pesticide

VectoBac WDG is a water-dispersible granule formulation containing 3,000 International Toxic Unit/mg of *Bti* (strain AM 65-52). Containing only *Bti* and food-grade inert ingredients, it has been approved for use in organic crops and sensitive habitats around the world. We chose it for this study because of the favorable environmental profile, safety for non-target organisms, and fast results; all mortality usually occurs within 12–72 hrs. The product is mixed by weight with tap water. The US product label allows for application rates of 123–988 g/ha for ground spraying equipment.

### Equipment

We tested three sprayers: a Cougar ULV cold aerosol generator (Clarke, Roselle, IL), a CSM2 Mist Sprayer (Buffalo Turbine, Springville, NY), and an Ag-Mister LV-8 low volume sprayer (Curtis Dyna-Fog, Westfield, IN). We chose the Cougar to examine the feasibility of using conventional ULV equipment with WDG. The standard pump was replaced with an optional QB3CSC 1/2 inch pump (Fluid Metering, Inc., Syosset, NY) to increase flow rate. However, despite being rated for a flow rate of over 2 L/min, we were unable to achieve more than 1 L/min. That flow rate was too low to reach our lowest application rate of 400 g/ha and the Cougar was not evaluated further.

The Buffalo Turbine Mist Sprayer (CSM2) has a 189 L stainless spray tank. A mechanical twin piston pump sends fluid to a spray head contained within an air chute. A gas-powered wind turbine propels the fluid from the spray head. The standard spray head consists of four flat-fan spray nozzles. For this study, we replaced the standard head with an optional Monsoon gyratory atomizing spray head (Buffalo Turbine, Springville, NY) for increased flow rate and more precise control over the droplet spectra. With the atomizing head, fan blades cause the head to rotate in relation to the wind speed. Fluid released from the center of the head is forced through stainless mesh screens; larger mesh producing larger droplets. We evaluated 0.33, 0.51, and 0.91 mm screens. The smallest screen was too fragile to be used alone and was supported with the 0.51 mm screen. Pump pressure was set to 1,034 kPa and a ball valve on the pump outlet was adjusted to a flow rate of 8.3 L/min. Wind speed was controlled with the engine throttle and set at maximum (177 km/h) for all tests for greatest swath width.

The LV-8 is a low volume sprayer designed for orchard spraying. An electric pump pushes liquid to eight nozzles arranged in two vertical rows of four. A gas-powered roots type blower generates air pressure to atomize the pesticide. A ball valve adjusts the flow rate on the fluid supply line and was set to 8.3 L/min. Air pressure was adjusted by changing engine speed. Pump pressure was maintained at 68.9 kPa. As equipped from the factory, the nozzles point out to the sides of the spray truck and may not release droplets high enough to make it over buildings and other obstacles. Given the narrow streets in many urban neighborhoods, the LV-8 may end up applying material directly to cars parked along the streets. To minimize this occurrence, we cut and welded the booms containing the nozzles to angle at 45 degrees so that the spray would be released over parked cars.

### Droplet Characterization

We characterized the droplet spectra at the Navy Entomology Center of Excellence (Jacksonville, FL) with a 2-D Phase Doppler Particle Analyzer system (TSI Inc., Shoreview, MN). The transmitter and receiver were outfitted with 500 and 300 mm lenses, resulting in a measurable droplet size range of 0.6–211 µm respectively. Equipment was positioned 1.2 m from the lens array. We measured the droplet size at the horizontal center of the spray scanning the whole plume vertically in a continuous mode and characterized the droplet spectra under a variety of flow rates and pressures with three replicated measurements recorded for each test.

We set all equipment for a flow rate of 8.3 L/min. The fluid pump of the LV-8 was therefore set at 68.9 kPa for each test and we adjusted the engine speed to change the air pressure through the nozzles, thereby varying the droplet size. With the CSM2 the fluid pump pressure was maintained at 1,034.2 kPa for all tests. Finally, we kept the Buffalo turbine wind speed at a maximum of 177 km/h for greatest swath width and varied droplet size by changing screen sizes on the atomizing spray head. VectoBac WDG was mixed with tap water at a concentration of 59 or 118 g/L with an electric paint mixer in 19 L plastic buckets. Target application rates were 400 and 800 g/ha (representing mid and high label rates on the USA pesticide label) assuming a swath width of 91.4 m and an application speed of 8 km/h.

### Open-field Trials

We conducted open-field trials to determine efficacy, swath width and droplet deposition under ideal conditions. The LV-8 was tested on private land over an open grass field at Arnold Groves Ranch (Clermont, FL) in September 2010 with permission from the property owner, Robert Arnold. The CSM2 was tested over a blacktop tarmac at the Trenton-Mercer Airport (Ewing, NJ) in October 2010 with permission from the Airport Manager, Melinda Montgomery. We set up stations in a 3×8 grid out to 76.2 m for the LV-8 and a 3×9 grid out to 91.4 m for the CSM2. The grids were set up parallel to the prevailing wind. Each station contained an empty 500 ml polyethylene container for larval bioassays and one Kromekote C1S card (Mohawk Fine Papers, Inc., Cohoes, NY) on a plastic compact disc case (for ballast) at ground level to determine droplet deposition and characteristics.

Sprayers were mounted on pickup trucks and driven at 8 km/h perpendicular to the wind during applications. We mixed the WDG with tap water at 59 and 118 g/L for the 400 and 800 g/ha rates respectively. A 2% solution FD&C Red 40 granular dye (Glanbia Nutritionals, Carlsbad, CA) was added to the mixture for droplet analysis. We operated the LV-8 at 62 kPa and 8.3 L/min with a volume mean diameter (VMD) of 107 µm from the nozzles. The CSM2 was operated with a 0.51 mm screen at 1,034 kPa and 8.3 L/min with a VMD of 233 µm from the spray head with the CSM2 head positioned at a 60° angle from horizontal. We made control applications with dye and water (CSM2) or water only (LV-8). Applications were made when wind speeds were between 3.2 and 8 km/h and stations were moved as needed to remain parallel to wind direction. We retrieved cards and cups 15 min after the applications allowing time for droplets to settle. Lids were placed on the cups to prevent contamination or loss of product. We replicated each application three times and performed one control application with each machine. Cups were returned to the laboratory for analysis where we added 400 ml water and 10 (CSM2) or 20 (LV-8) 3^rd^ instar *Ae. albopictus* larvae to each cup. Mortality was recorded at 24, 48 and 72 hours and final mortality rates were corrected for control mortality using the Schneider-Orelli formula [24]. We analyzed the Kromekote cards with the DropVision AG system (Leading Edge Associates, Inc., Waynesville, NC) a system that uses a portable scanner to digitize droplet cards. Stains on the card were counted and measured and the software provided droplet and volume data using user-defined application and mix rates.

### Operational Trials

We developed operational field trials in Trenton (Mercer County), New Jersey, USA (40° 12′ N, 74° 44′ W) and Belmar (Monmouth County), NJ USA (40° 13′ N, 74° 44′ W). The two Trenton sites were urban residential neighborhoods: S. Olden (40° 22′ N, 74° 73′ W) was 48.6 ha consisting of 24 residential blocks, each containing a residential street on all four sides, sometimes divided lengthwise by a drivable alley. St. Olden included 1,250 parcels (i.e. house with surrounding yard) most often built as adjoining row homes or duplexes [25]. Parcel sizes were relatively constant at approximately 200 m^2^. The second site, Cummings (40° 21′N, 74° 74′W), was 30 ha with 23 residential blocks, occasionally divided lengthwise by a drivable alley. Cummins included 1,122 parcels many adjoining (i.e. row houses). Almost all adjoining parcels contained a sheltered alcove area between homes, where vegetation and trash proliferated creating mosquito resting areas. Socioeconomic conditions in both St. Olden and Cummins have led to a large number of abandoned homes with neglected yards that accumulate containers such as buckets, cans and other trash that can hold water [26].

The field site in Belmar was a seaside resort community located along the Atlantic coast and consisting mainly of traditional suburban single- family home parcels with yard space on all four sides, interspersed with seasonal rental properties. A small section of the plot contained commercial storefront and several large condominiums and motels. This 121.9 ha site consisted of 59 residential blocks and 2,356 parcels averaging 0.1 ha in size. The socioeconomic conditions of the site can best be described as upper-middleclass. The majority of the homes are well maintained and container mosquito habitat, such as planters and flexible downspout extensions, tend to be cryptic but abundant. Rental properties often had more traditional container habitats such as buckets, toys, and recyclable containers.

We used either a LV-8 or a CSM2 mist sprayer for each application. We set up the equipment as previously described for a flow rate of 8.3 L/min. We mixed VectoBac WDG with tap water at a rate of 59 or 118 g/L and drove the vehicles at an average speed of 8 km/h for a final application rate of 400 or 800 g/ha. Meteorological data was recorded for wind speed, direction, humidity, and temperature at 1 m and 10 m heights for monitoring thermal inversion. A Vantage Pro2 wireless weather station (Davis Instruments, Hayward, CA) was set up within the treatment site 14 h prior to application and maintained until 8 h post application. We made applications from April through September 2011, June 2012, and July 2013 between 01∶00 and 06∶00 hours. All pesticide applications were made by county mosquito control agencies under the authority of Title 26∶9 of the New Jersey Administrative Code.

We selected 30 residential parcels within our treatment sites and 10 parcels within a control site in either Mercer or Monmouth for placement of bioassay cups. Within each parcel, dry bioassay cups were placed in front, alongside, and in the backyard of each home. A total of 210 polyethylene 500 ml bioassay cups per replicate were placed in the field 2 hrs prior to application and removed 1 hr post application to allow time for droplets to settle into cups. Bioassay cups were used in lieu of sampling natural larval populations to get consistent and comparable data points. Existing larval habitats were too transient to be of statistical value and sampling of varied containers could not be standardized.

All cups were transported to the laboratory and loaded with 400 ml of distilled water and 40 mg of a 50∶50 mix of yeast:lactalbumin (Sigma-Aldrich, St. Louis, MO) added as an emulsion in 1 ml of water as a food source for larvae. Then we added 10–15 late 2^nd^ to early 3^rd^ instar *Ae. albopictus*. Mortality was recorded at 24, 48, and 72 hrs post-application. Mortality rates were corrected for control mortality using the Schneider-Orelli formula [24].

Based on the outcome of the applications, we selected three of the operational trials to determine the residual efficacy of the treatments. For these experiments, we retained any cups which achieved 100% mortality after 72 hours. Dead larvae were removed from the cups and ten early 3^rd^ instar *Ae. albopictus* were added. After 72 hours mortality was recorded, dead larvae were removed, and ten fresh larvae were added. We repeated this process until larval mortality dropped to or below 20%.

### Statistical analyses

All mortality data were reported as proportions and arcsin square root transformed to normalize the distributions [27]. After the open field trials we performed least squares repeated measures analyses of variance in JMP (SAS Institute, Cary, NC) to examine the effects of equipment type and application rates on mortality rates. As mentioned, there were three spatial replicates at increasing distances from the application site and each trial was replicated 3 times. To assess the reliance of the results on expectations of homoscedasticity of the data (hard to assess with a feasible number of replicates), we also performed the repeated measures analysis with ranked data.

After the operational field trials, we examined the effects of equipment type, application rate and location of bioassay containers (front, middle, and back) with a least squares analysis of variance after confirming normality of the distributions using a goodness of fit test in JMP (SAS Institute, Cary, NC).

## Results

### Droplet Analysis

Unlike ULV sprayers that are optimized to produce droplets below 30 µm, the LV-8 is designed to produce larger droplets for agricultural use. Under laboratory conditions, median droplet sizes ranged from 74–141 µm (Table 1). In general, increasing air pressure led to larger droplet sizes with the LV-8. Beyond 34.5 kPa there was a direct relationship between air pressure and droplet size. With the CSM2, VMD’s ranged from 214–233 µm.

**Table 1:** Droplet spectra for VectoBac WDG determined by 2-D phase doppler particle analyzer ± SEM.*

Equipment	Setting	DV_0.1_ (µm)	DV_0.5_ (µm)	DV_0.9_ (µm)
**LV-8**	34.5 kPa	35.7±2.2	140.7±22.4	276.0±13.0
	41.4 kPa	31.5±0.5	73.5±1.5	202.5±0.5
	42.7 kPa	34.0±0.6	81.0±6.2	217.0±26.0
	63.4 kPa	28	92	250
	65.5 kPa	34.7±0.9	109.7±9.7	262.0±6.4
**CSM2**	0.33 & 0.51 mm screen	71.3±8.2	214.0±11.5	313.7±6.6
	0.51 mm screen	82.0±3.1	232.7±2.4	324.7±2.4
	0.91 mm screen	64.3±9.2	218.0±10.1	313.3±16.2

*VectoBac WDG mixed with tap water at 59 g/l. Equipment set for final application rate of 400 g/ha.

### Open-Field Trials

**Figure 1:**
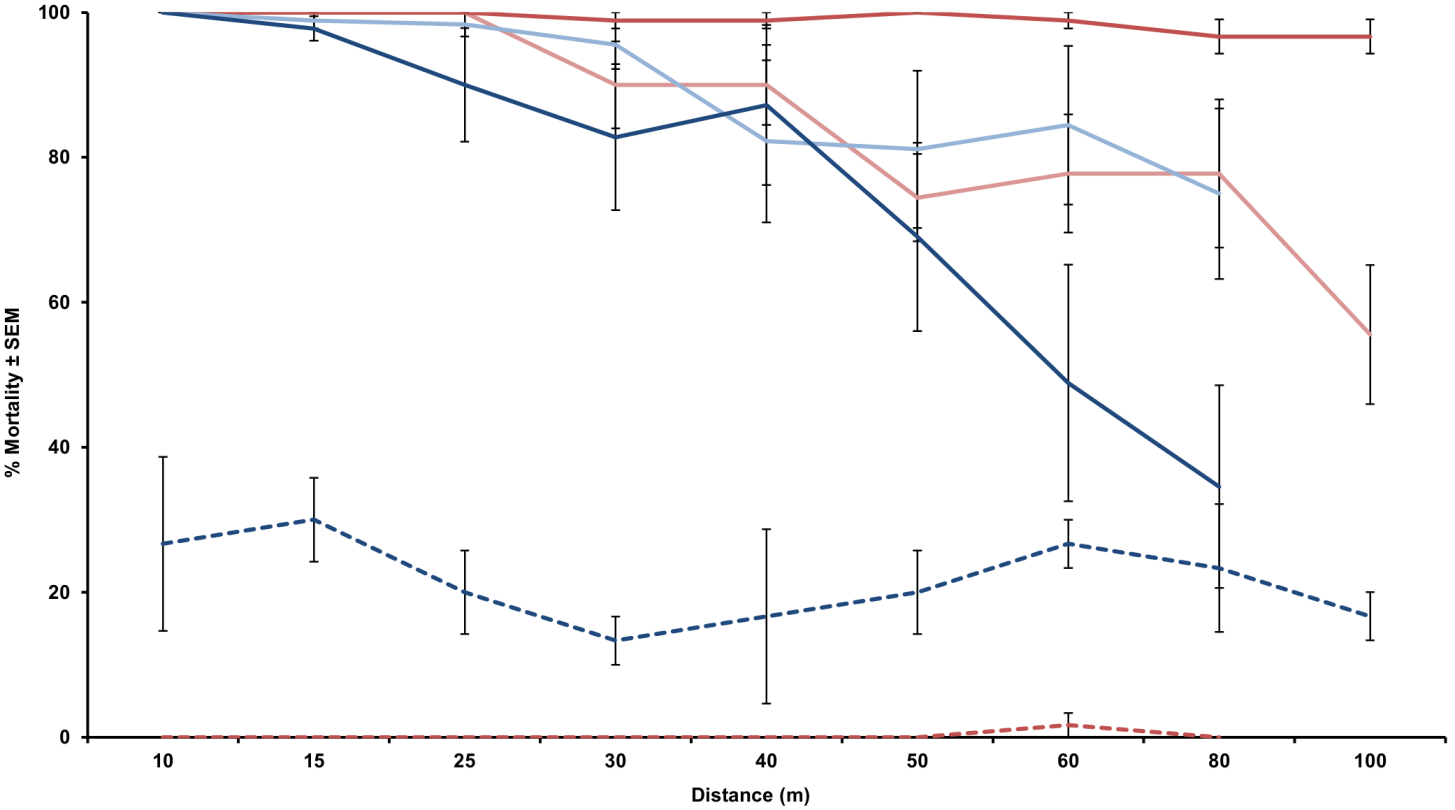
*Aedes albopictus* 72 hr mortality rates for VectoBac WDG applied with a CSM2 and LV-8 at 400 and 800 g/ha in an open-field. Red = CSM2, blue = LV-8, light colors = 400 g/ha, dark colors = 800 g/ha, dashed line = control.

Weather conditions during the LV-8 tests averaged 23.9°C, 85% RH and 4.8 km/h wind speed. Average conditions during the CSM2 runs were 14.4°C, 55% RH and 3.2 km/h wind speed. The LV-8 resulted in an average mortality of 89.4±6.4 and 76.3±9.2% out to 80 m (maximum distance tested) for the 400 and 800 g/ha rates, respectively. The CSM2 averaged 98.9±0.9% mortality out to 100 m at the 800 g/ha rate and 85.1±5.1% at the 400 g/ha rate (Fig. 1). There was no significant difference in the overall average mortality at each distance from deployment between rates or machines. However, when only data for the CMS2 is examined, the average mortality was greater at the 800 g/ha rate than at the 400 g/ha rate (*P* = 0.04) (Fig. 1). Repeating the analysis with ranked data yielded the same results.

**Figure 2:**
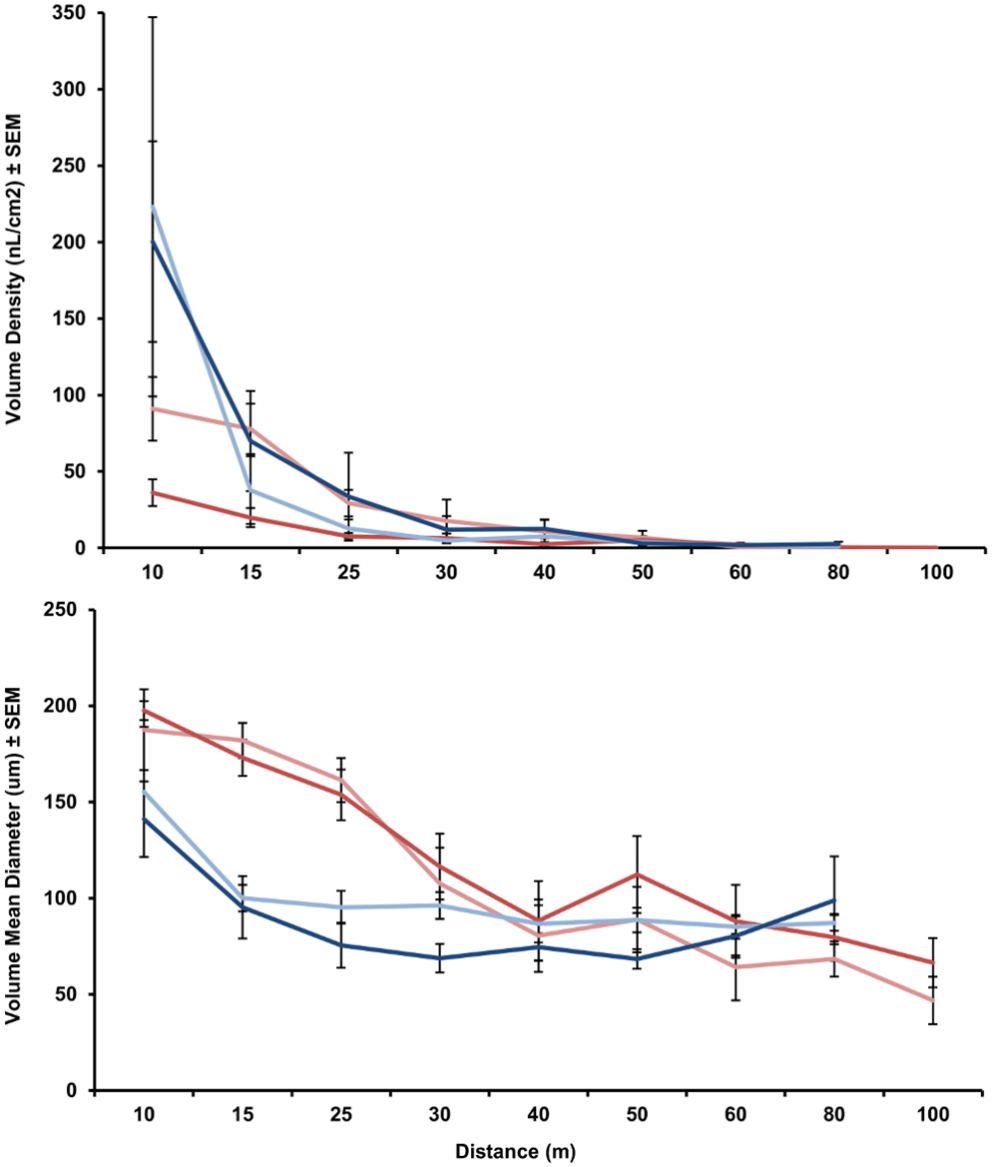
Volume density and droplet sizes of VectoBac WDG applied with a CSM2 and LV-8 at 400 and 800 g/ha in an open-field. Red = CSM2, blue = LV-8, light colors = 400 g/ha, dark colors = 800 g/ha.

We conducted a droplet analysis to determine droplet size, swath width and deposition rates (Fig. 2). The LV-8 had greater deposition at the 10 m stations than the CSM2. At distances beyond 10 m, there was little difference in the deposition rates regardless of equipment or application rate. Minimal deposition at the farther distances still resulted in significant mortality (Fig. 1). Application rates were determined by the mix ratio alone. The 800 g/ha rate resulted in greater volume deposition for the LV-8 but lower deposition for the CSM2. Generally, larger droplets settled out closer to the spray line and decreased swath width (Fig. 2). There was a clear correlation between VMD and distance for the CSM2 (average r^2^ = 0.9) while the droplets from the LV-8 appear to be relatively uniform across the measured swath width.

### Operational Trials

**Figure 3:**
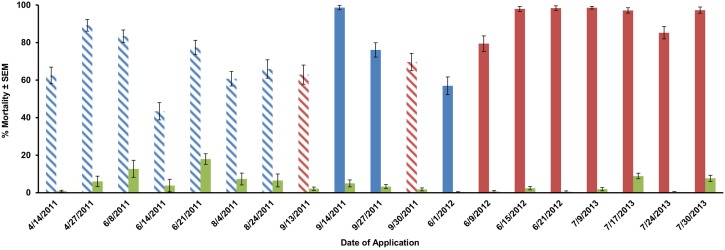
Efficacy summary of area-wide VectoBac WDG applications by date. Red = CSM2, blue = LV-8, green = control, dashed lines = 400 g/ha, solid lines = 800 g/ha.

The efficacy of control in urban and suburban neighborhoods in 2011 were mixed with an average efficacy of 72.0±1.3%. In contrast, by the end of 2012 we were able to consistently achieve high mortalities in urban settings (over 91%), which we further increased in 2013 to 94.5±1% (Fig. 3). However, operational results varied greatly for the LV-8. Of the ten applications made with the LV-8, we were only able to exceed 80% mortality on three occasions. In contrast, the CSM2 averaged 89.4±1.0% mortality over all nine applications and exceeded 90% mortality for five of those applications.

**Figure 4:**
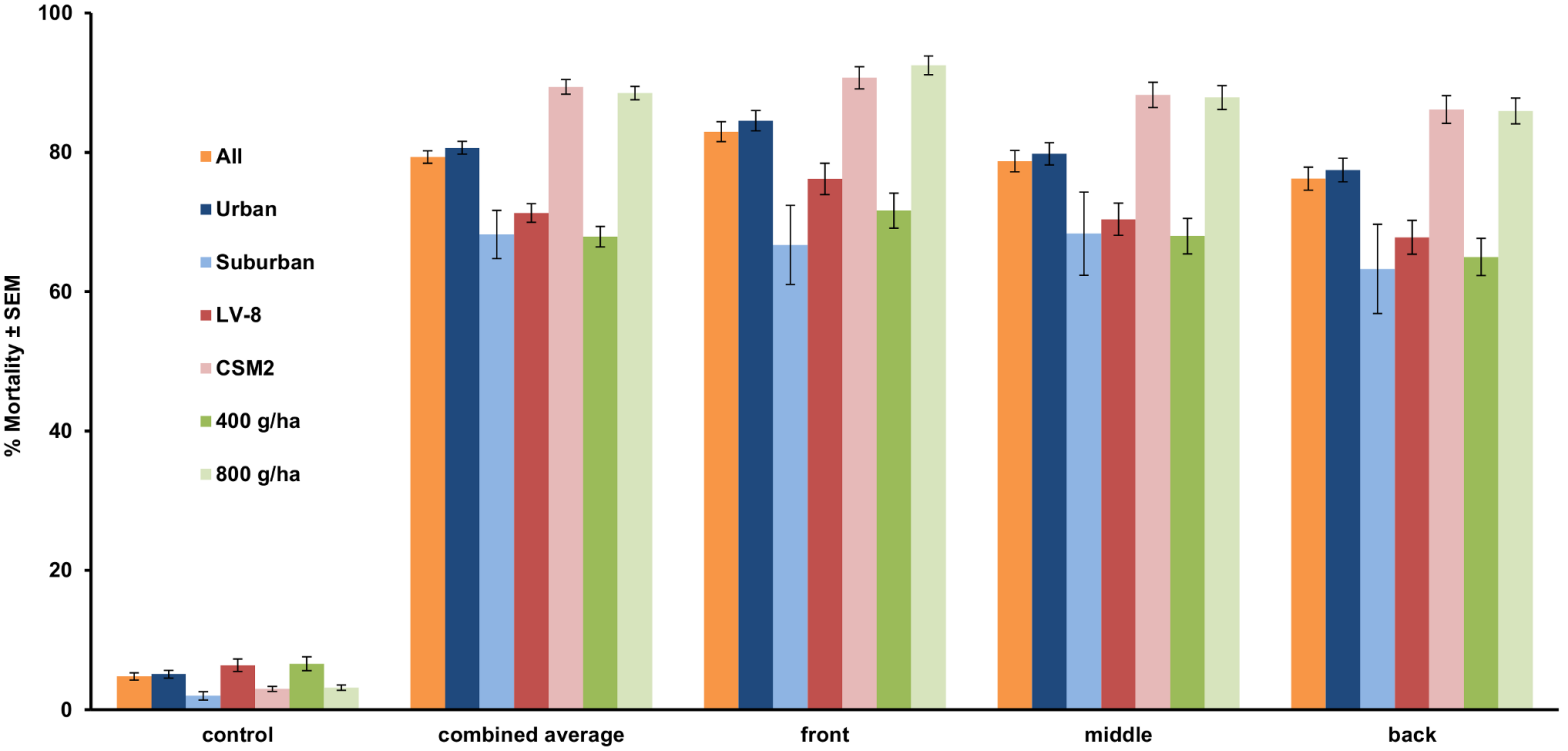
Efficacy by location of VectoBac WDG applied with a CSM2 and LV-8 at 400 and 800 g/ha in residential areas. Orange = average efficacy, dark blue = urban plots, light blue = suburban plots, red = LV-8, pink = CSM2, dark green = 400 g/ha, light green = 800 g/ha.

**Figure 5:**
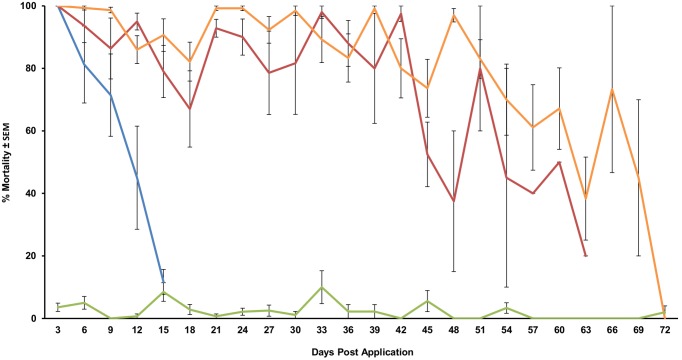
Residual efficacy of VectoBac WDG applied with a CSM2 and LV-8 at 400 and 800 g/ha in residential areas. Orange and red = CSM2 at 800 g/ha, blue = LV-8 at 400 g/ha, green = control.

Although we had slightly greater success in urban (Mercer) than in suburban areas (Monmouth) (Fig. 4), we only performed two applications in suburban Monmouth and both were performed with the CSM2 at the 400 g/ha rate. Since these were the only operational trials with the CSM2 at the lower rate we did not include these two suburban trials in the statistical analysis. Despite a slight decline in mortality as distance from the spray line increased, operational mortality rates were similar across all cup locations and the differences were not significant (*F_2,2_* = 1.31, *P* = 0.28). We did however see significantly greater efficacy at the higher application rate of 800 g/ha (*F_1,1_* = 4.26, *P* = 0.04), and the CSM2 outperformed the LV-8 (*F_1,1_* = 8.64, *P* = 0.005). Under laboratory conditions, the residual activity of the applications achieved over 80% mortality for 6 weeks and lasted nearly 10 weeks (Fig. 5).

## Discussion

Unlike most pressurized spray equipment where air pressure has an inverse relationship with droplet size, the LV-8 exhibited a direct relationship between air pressure and VMD. It is unclear if this trend was a result of nozzle design or was an artifact of the properties of the liquid WDG, which is an emulsion rather than a solution. However, the largest droplets did occur at the lowest pressure setting indicating that the nozzles were not operating within their optimal range when spraying the viscous mixture of WDG. Also of note, screen size did not seem to have a predictable effect on droplet size for the CSM2. It is plausible that running the head at maximum rpm canceled out any effect from the screens by creating additional wind shear. Reducing turbine speed may result in different droplet sizes but will reduce swath width. We only tested the 59 g/L mix ratio for the particle analyzer tests since existing literature [28] demonstrated that there was little advantage to application rates above 400 g/ha.

Based on equipment design, the droplets from these mist blowers were significantly larger than the droplets created by a ULV machine applying the same material [29]. Similar studies with back pack mist blowers produced droplets comparable in size to the LV-8 [22]. The insufficient flow rates we experienced with the modified Clarke Cougar suggest that most ULV sprayer will be unsuitable for WDG application without extensive modification.

In open field trials we observed the expected decrease in mortality with increasing distance from the spray line regardless of equipment or rate; however the CSM2 outperformed the LV-8 in both swath width and efficacy. The LV-8 had greater deposition at the 10 m stations than the CSM2 indicating that larger drops settled out earlier likely because the LV-8 is a passive sprayer (i.e. it does not induce a strong directional force to the droplets the way the CSM2 does). The wind speed generated by the turbine of the CSM2 propelled the large droplets further, resulting in a more uniform deposition rate. The performance of the LV-8 was more dependent on wind speed than the CSM2: for example, with the LV-8, the 800 g/ha rate did not perform as well as the 400 g/ha rate probably because of environmental conditions as the wind averaged 4.8–9.6 km/h for the 400 g/ha trials and only 0–3.2 km/h for the 800 g/ha trials. While the CSM2 was less dependent on environmental conditions, the wind from the turbine pushed droplets beyond the closest stations, resulting in lower deposition rates near the spray vehicle. Importantly, we observed very high toxicity of WDG to mosquito larvae such that the lower deposition rates did not result in lower mortality.

Mortality in the controls was low for the LV-8 but significant mortality was observed in the CSM2 controls. This is likely because the LV-8 is an easier machine to clean and load and had been used for orchard spraying of agricultural pests with agricultural pesticides, not mosquito larvicides. In contrast, the CSM2 had routinely been used in a mosquito control program for several years, exclusively with mosquito larvicides. Further, on the LV-8 the removable spray tank is translucent and the fluid lines are transparent, making it easy to see when the unit has been sufficiently cleansed. The spray tank on the CSM2, is stainless steel and is welded into the machine. The tank is filled through a 51 mm diameter neck and emptied through a 19 mm drain plug. Flushing the tank is difficult and there is no way to see inside the tank to determine when the tank is clean. The fluid lines also have metal fittings that corrode and provide recesses for pesticide residue to collect. Despite repeated efforts to clean the CSM2 tank, residual material may have persisted during the control applications. We have since also conducted experiments to confirm that the food dye used in the controls was not toxic to *Ae. albopictus* at the rates applied [30].

The VMD values at the 10 m stations suggest that the droplet data from the spray cards are reliable as they are close to the VMD values from the nozzle as determined by the laser analysis. While it is logical to presume that the droplets from the Buffalo Turbine are too large (VMD = 233 µm) to travel 100 m, the 177 km/h wind created by the turbine was sufficient to push the drops, compensating for the extra mass of the large drops. The smaller drops from the LV-8 (VMD = 107 µm) were still small enough to drift out to 80 m from wind currents alone. We did not find significant differences in droplet size based on the mix ratio of the product which contradicts previous studies that found that higher mix ratios resulted in larger droplets [22]. This is likely because of the low power of the backpack mist blower used in that study. The more powerful motors of the truck-mounted machines we used appear better equipped to handle the more viscous mix. The problem with many water-based formulations is the evaporation of droplets. Personal communication from Valent technical staff (Peter DeChant, 21 July 2010) indicated that WDG will not evaporate beyond a terminal droplet size of around 80 µm. Our data support this assertion. Regardless of the initial size, beyond 40 m droplet sizes converged to an average VMD of 80.7 µm.

Smaller droplets are lighter than larger droplets and therefore should drift farther yielding greater swath width. Other open-field trials with ULV sprayers produced small droplets of 20–50 µm yet a swath of only 30 m [31]. These sprayers are normally used for mosquito adulticides where small drops are expected to drift up to 90 m without settling. It is possible that many of the droplets created by the ULV sprayer in Lee et al. [31] drifted beyond the sample area. For the purposes of applying mosquito larvicides, larger droplets are preferable so that they settle into containers before drifting past the target area. Based on our results we recommend that equipment should be optimized for larger droplets (≥80 µm).

We expected a positive correlation between mortality and volume density, higher volume density resulting in greater mortality. However, the LD_50_ values for WDG against *Ae. albopictus* are so low, 3 ppb as determined against our laboratory strain, that even a single droplet in the bioassay cups was sufficient to cause 100% mortality. Any deposition over 18 nL/cm^2^ resulted in 100% mortality and in many cases rates below 1 nL/cm^2^ were still able to provide 100% mortality. While there is no correlation between volume density and mortality, the data demonstrate that equipment can be calibrated without bioassays. As long as droplets are visible on spray cards, mortality should be acceptable at that distance.

The open-field trials tested area-wide larval control from truck-mounted equipment under ideal conditions in the absence of any obstructions. Operational conditions present many obstacles to any type of space spray. Houses, automobiles, and vegetation all serve to potentially impede the drift of pesticide sprays. To evaluate the operational utility of this technique, we conducted operational applications under operational conditions. These data show a clear improvement in our area-wide technique over the three seasons of the study. Early in the study we focused on a 400 g/ha rate from the LV-8. Equipment problems such as pump failure, solenoid malfunctions, and short circuits of the electrical system led to poor results in several of the early trials. Further, contrary to what we found in the open field trials, bioassay results indicated that the 400 g/ha rate was not delivering enough material into distant containers. In late 2011 we increased the rate to 800 g/ha and found that this change yielded improved yet inconsistent results from the LV-8. In 2012 and 2013 all applications were made at the 800 g/ha rate and all but one application was made with the CSM2. This combination of higher application rate and better machine led to high mortality rates in all trials.

Within each parcel, the consistent results of the operational trials demonstrate that this technique can be used effectively in any type of residential environment with different types of equipment. The WDG spray effectively traveled around obstructions and settled in containers. In total, larval mortality was observed in 90% of the treatment cups despite efforts to place cups in inaccessible areas such as under decks and in dense vegetation. Our results are limited to bioassay cups but we expect similar results in other habitats such as roof gutters although these areas were not monitored. Additionally, the observed persistence of WDG indicates that rainfall following WDG applications would likely wash *Bti* to non-target areas such as catch basins and sump pumps leading to increased control.

As a result of the thorough coverage, there was no significant difference in mortality based on the location of the cups. Bioassay cups placed in the backyards of homes did just as well as cups placed in front of homes nearest the spray line. This is similar to Jacups et al. [22] using a backpack mist blower over various distances. However our truck mounted strategy has the advantage of being more consistent and keeps the operators inside the truck cab away from the *Bti* spray.

The greater efficacy we observed in the urban areas was not a result of the smaller parcel sizes or easier access in those plots, as there was no difference in efficacy across distances even in the suburban parcels. Instead, it is more likely due to the fact that the suburban trials were only run at the 400 g/ha rate at a time when we thought based on the results of the open-field trials, and the results of Jacups et al. [22] in a bushland setting, that the higher application rate was unnecessary. Obstacles present during operational trials likely resulted in lower deposition rates which would explain the increased mortality at the higher application rate. We were unable to measure deposition during operational trials because we could not apply dyed material in a residential setting. High environmental humidity precluded the use of water sensitive paper and ultraviolet dye did not mix well with the WDG solution. However, it is logical to conclude that deposition rates would decrease given the physical obstacles present under operational conditions. With fewer droplets reaching the cups, the amount of *Bti* in each droplet becomes increasingly critical especially at the farthest distances. Significantly, our application rate was below the maximum of 988 g/ha specified on the product label. Higher application rates could provide even greater mortality, although as discussed mixing and spraying at high rates has limitations.

The CSM2 performed significantly better than the LV-8 overall most likely because the LV-8 is a passive machine relying on wind to carry the spray cloud. Wind conditions were less than optimal for most applications, rarely exceeding 4.8 km/h, putting the LV-8 at a disadvantage. By contrast the turbine of the CSM2 generates its own wind and is therefore less reliant on environmental conditions. This is further evidenced by the consistency of the CSM2 across all distances. It is possible, however, that under more favorable wind conditions, the efficacy of the LV-8 would be higher.

Although not considered a residual product, *Bti* has been shown to give extended control in containers [32]–[35]. Our applications achieved over 80% mortality for 6 weeks and lasted nearly 10 weeks. It is important to note that the residual trials were conducted in the laboratory. While environmental conditions could reduce the residual activity of *Bti*, trials conducted under semi-field conditions have provided residual control for up to 11 weeks [22]. In that study, WDG was applied closer to the containers with a backpack mist blower leading to droplet densities up to 300 drops/cm^2^. In our open-field trials, droplet densities averaged 10 drops/cm^2^ and never exceeded 81 drops/cm^2^ and Jacups et al. [22] found that higher application rates resulted in longer residual control. Given that there were significant differences in mortality between machines and rates, it is likely that the reduced residual efficacy of the LV-8 application at 400 g/ha was due to fewer *Bti* particles reaching the containers.

Based on our open-field and operational trials, both sprayers are suitable for area wide larval control operations. The CSM2 offers the advantages of higher potential flow rates, adjustable nozzle angle, and increased swath width due to the 177 km/h wind drift created by the turbine. However, capacity is limited to 189 L (about 25 min of spray time), and the hurricane force wind from the turbine may present problems in a residential areas rattling windows, blowing over potted plants, scattering debris, etc. The LV-8 has the advantage of an optional 568 L spray tank with an onboard mixer, which increases the potential run time and makes loading the WDG easier. However, as discussed the LV-8 is a passive sprayer that relies on the wind to carry the pesticide to the target areas. Wind speeds often averaged less than 4.8 km/h during our operational trials, a standard condition in very urban areas at night, leading to inconsistent efficacy with LV8-applications. With the reconfigured nozzles, we found that WDG deposition to parked cars was minimal and similar to what was left by the CSM2. Observations by the team indicate that applications left a fine dust on automobiles not easily distinguished from dirt or pollen. The WDG rinsed easily from vehicles and caused no damage to automotive paint. Although citizens were in regular contact with the mosquito control programs, we received no negative comments from the public regarding visible residues from any of the 19 area-wide applications over 3 years.

The present study indicates that area-wide applications of larvicides with truck-mounted equipment have the clear potential to reduce *Ae. albopictus* populations in urban and suburban residential areas by killing immature mosquitoes in open containers. Both larvicide doses effected high larval mortality that could be sustained for over one week and high dose applications exhibited up to 90 days of residual control. Street-level applications were able to drift past obstructions and settle into containers located up to 100 m away with no loss of efficacy at greater operational distances. In conclusion, we provide evidence that a nighttime LV application of VectoBac WDG can be efficacious in killing larvae of *Ae. albopictus* in urban and suburban neighborhoods.

It is important to note, however, that effective larval control does not necessarily translate into a reduction in the adult mosquito population or human landing rates. In fact, a reduction in larval population can actually result in an increase in adult emergence for some species [36], but this does not appear to be the case with *Ae. albopictus* [37]. We deployed a high-density of BG Sentinel (Biogents AG, Regensburg, Germany) mosquito traps throughout each operational research plot for the duration of the study and have measures of adult populations of *Ae. albopictus.* These data were not presented here because we were performing multiple control strategies simultaneously, as needed operationally to safeguard the well being of the residents. Those data are being developed into a separate publication. In brief, we found that when area-wide applications of WDG were coordinated with a degree-day model and traditional adulticide spray missions, there was a synergistic effect resulting in a significant decrease in the adult *Ae. albopictus* populations.

This study was conducted with readily available equipment adapted for larvicide applications. Purpose-built equipment could provide even better efficacy and easier operational use. New formulations of *Bti* developed for this technique that mix with water more easily or come pre-mixed would greatly reduce the labor involved in preparing and loading the product. Future studies are needed to determine the optimal timing of applications so as to have maximum impact on adult mosquito populations. Based on our findings, we recommend that nighttime applications of LV larvicides in areas with large *Ae. albopictus* populations be considered as part of an integrated approach for public health protection.
